# The influence of electrocardiogram-gated computed tomography reconstruction into 8 or 10 cardiac phases on cardiac-pulsatility-induced motion quantification of stent grafts in the aorta

**DOI:** 10.1016/j.jvssci.2023.100131

**Published:** 2023-09-28

**Authors:** Jaimy A. Simmering, Deborah A. Zagers, Robert H. Geelkerken, Henny Kuipers, Gerben A. te Riet o.g. Scholten, Michel M.P.J. Reijnen, Cornelis H. Slump

**Affiliations:** a Division of Vascular Surgery, Department of Surgery, Medisch Spectrum Twente, Enschede, the Netherlands; b Multi-modality Medical Imaging (M3i) Group, Faculty of Science and Technology, Technical Medical Centre, University of Twente, Enschede, the Netherlands; c Robotics and Mechatronics (RaM) Group, Faculty of Electrical Engineering, Mathematics and Computer Science, Technical Medical Centre, University of Twente, Enschede, the Netherlands; d Department of Surgery, Rijnstate, Arnhem, the Netherlands

**Keywords:** ECG-gated computed tomography, Endovascular procedures, Aorta, Stent graft, Dynamic

## Abstract

**Objective:**

The goal of this study was to determine to what extent aortic stent graft motion quantification is comparable between electrocardiogram (ECG)-gated computed tomography (CT) scans with reconstructions into 8 and 10 cardiac phases on CT scanners from two different vendors.

**Methods:**

An experimental setup that induces motion of an aortic stent graft, according to a predefined aortic blood pressure wave, was placed in two CT scanners of different vendors. The stent graft motion was captured using an ECG-gated CT technique and quantified using dedicated analysis algorithms. The calculated motion amplitudes and total traveled path lengths of stent segmentations were compared between scans reconstructed into 8 and 10 phases and between the scanners, after validation with sensor measurements and repeated measurements.

**Results:**

No difference in motion amplitudes in z-direction (craniocaudal direction) was observed between the reconstructions into 8 and 10 phases (0.02 mm; 95% confidence interval [CI], –0.01 to 0.05 mm; *P* = .358). The z-amplitudes differed by 0.04 mm (95% CI, 0.01-0.07 mm; *P* = .003) between the different CT scanners. Path lengths differed 0.07 mm (95% CI, 0.01-to 0.13 mm; *P* = .013) between the reconstructions into 8 and 10 phases and 0.13 mm (95% CI, 0.06-0.17 mm; *P* < .001) between the different scanners.

**Conclusions:**

The motion amplitudes can accurately be compared between 8 and 10 phases and between the two scanners, without differences larger than the voxel size of 0.3 × 0.3 × 0.5 mm. Clinical motion analysis results of different ECG-gated CT scans and CT scanners can be compared up to the accuracy of the CT scan.

## Clinical Relevance

This in vitro experiment showed that the motion amplitudes of stent grafts during the cardiac cycle can accurately be compared between electrocardiogram-gated computed tomography (CT) scans reconstructed into either 8 or 10 phases and between scanners of different manufacturers, without differences larger than the voxel size of 0.3 × 0.3 × 0.5 mm. In other words, clinical motion analysis of stent grafts using different retrospective electrocardiogram-gated CT scans and CT scanners of different manufacturers can be compared up to the accuracy of the CT scan.

**Article Highlights**

• **Type of Research:** in vitro study
• **Key Findings:** This in vitro experiment showed that the motion amplitudes of stent grafts during the cardiac cycle can accurately be compared between electrocardiogram (ECG)-gated computed tomography (CT) scans reconstructed into either 8 or 10 phases and between scanners of different manufacturers, without differences larger than the voxel size of 0.3 × 0.3 × 0.5 mm.
• **Take Home Message:** Clinical motion analysis of stent grafts using different ECG-gated CT scans and CT scanners of different manufacturers can be compared up to the accuracy of the CT scan.

The heart induces a pulse wave through the aorta 60 to 100 times every minute. Every heartbeat, this cardiac pressure wave induces suprarenal and infrarenal aortic expansion, ranging between 1 and 2 mm.^1^, ^2^, ^3^ Stent grafts developed for endovascular aneurysm repair (EVAR) have to withstand these repeated forces circa 30 million cardiac cycles per year,^4^ which in turn challenges the durability of EVAR on the long term. To investigate the pulsatile motion of stent grafts retrospective electrocardiogram (ECG)-gated computed tomography (CT) scanning is a useful tool. Retrospective ECG-gated CT scanning allows for 4 dimensional (ie, three-dimensional [3D] plus time) visualization and quantification of the aortic wall and stent graft motion during the cardiac cycle. This is achieved by simultaneously collecting the ECG and CT data and reconstructing CT volumes from raw CT data acquired during the predefined cardiac phases.^5^^,^^6^ Retrospective ECG-gated CT scans have been used to investigate motion and deformation of the aorta and its stent grafts during the cardiac cycle^1^, ^2^, ^3^^,^^7^, ^8^, ^9^, ^10^, ^11^, ^12^, ^13^ However, the number of cardiac phases into which the CT data was reconstructed varied between these studies as some had reconstructed the CT into 8 phases and others into 10 phases, rendering the comparability between studies uncertain. Moreover, as ECG-gated CT scans are becoming more and more frequent in both clinical trials and clinical workflow, the importance of accurate comparability among different scan protocols and CT scanners of different vendors increases further.

The objective of this study was to determine to what extent motion quantification is comparable for ECG-gated CT scans with reconstructions into 8 and 10 cardiac phases on CT scanners from two vendors in an experimental setup.

## Methods

### Experimental setup

**Fig 1:**
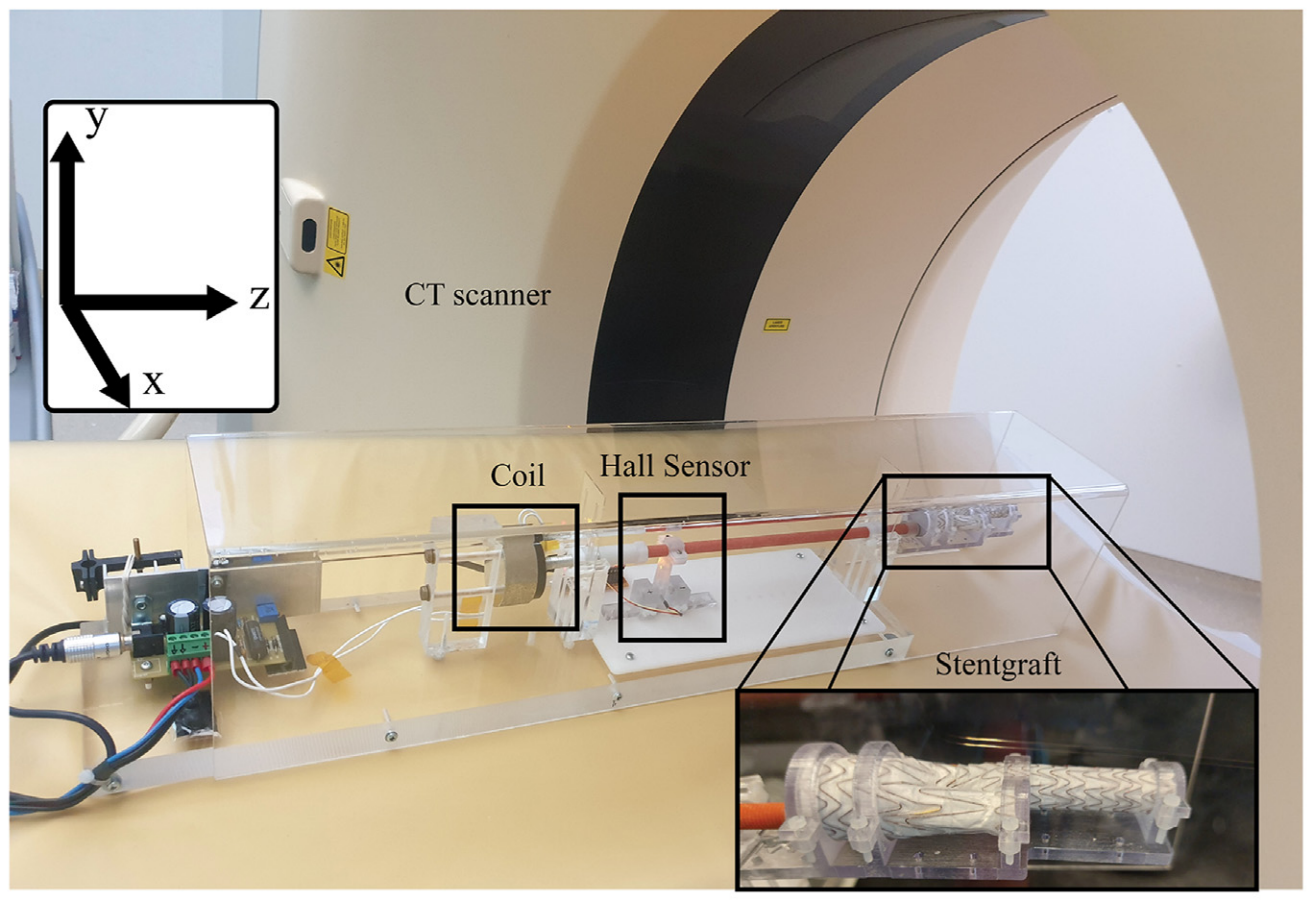
The experimental setup in a computed tomography (CT) scanner: The stent graft is moving in the z-direction (clinically the craniocaudal direction) of the scanner while attached to the red lever. This lever is stimulated by the triggered coil of the linear actuator. The hall sensor detects the true motion of system which can be interpreted using dedicated software.

An in-house developed linear actuator device was used to obtain a controlled motion in one direction (Fig 1). A Gore Excluder AAA Endoprosthesis (W. L. Gore & Associates, Inc., Newark, DE) was attached to the lever of the linear actuator and used as object for the ECG-gated CT imaging and motion quantification. The single direction motion of the linear actuator was induced by a custom made coil (linear voice coil actuator LA18-12-007Z, BEI Kimco, Vista, CA), with a maximal stroke of 3 mm back and forth (ie, maximal amplitude of 6 mm) that can be set by altering the voltage amplitude in the waveform generator. The actuator was stimulated by an Agilent HP 33,120A arbitrary waveform generator (Agilent Technologies, Santa Clara, CA) that was connected to an oscilloscope for visual feedback. An ECG simulator (ProSim 8 Vital Signs and ECG Patient Simulator, Fluke Biomedical, Everett, WA) was used as a synchronizing trigger for the CT scanner and the pulse generator. To obtain an accurate ground truth for the motion of the endoprosthesis, a Hall sensor (A1318LUA-2-T Hall Effect Sensor, 3-Pin SIP, Allegro Microsystems, Manchester, NH) was attached to the lever and centered between two magnets at the base of the linear actuator. The Hall sensor was controlled by an Arduino microcontroller (5V 16 M Mini Leonardo Microcontroller, Arduino, Turin, Italy) programmed with a specialized Arduino C-code. When the linear actuator moves the stent graft, the sensor produces the same displacement between the magnets. Based on the changing magnetic field that is experienced by the sensor, the motion of the stent was obtained. The sensor was calibrated by controlled relocation of the linear actuator, and thereby the sensor between the two magnets, with steps of 0.25 mm using a micrometer. This process was repeated once to ensure valid measurements. Through interpolation, measurements from the sensor were translated into distances of the stent with respect to the resting point. This calibration of the microcontroller before and after the experiment resulted in a Hall sensor measurement error of <0.1 mm. The sensor used a sample frequency of 100 Hz. The data from the sensor were read and saved with a specialized script in Processing (Processing 3.5.4 for Windows). Appendix A provides a more detailed description of the experimental setup.

### Generated motion patterns

**Fig 2:**
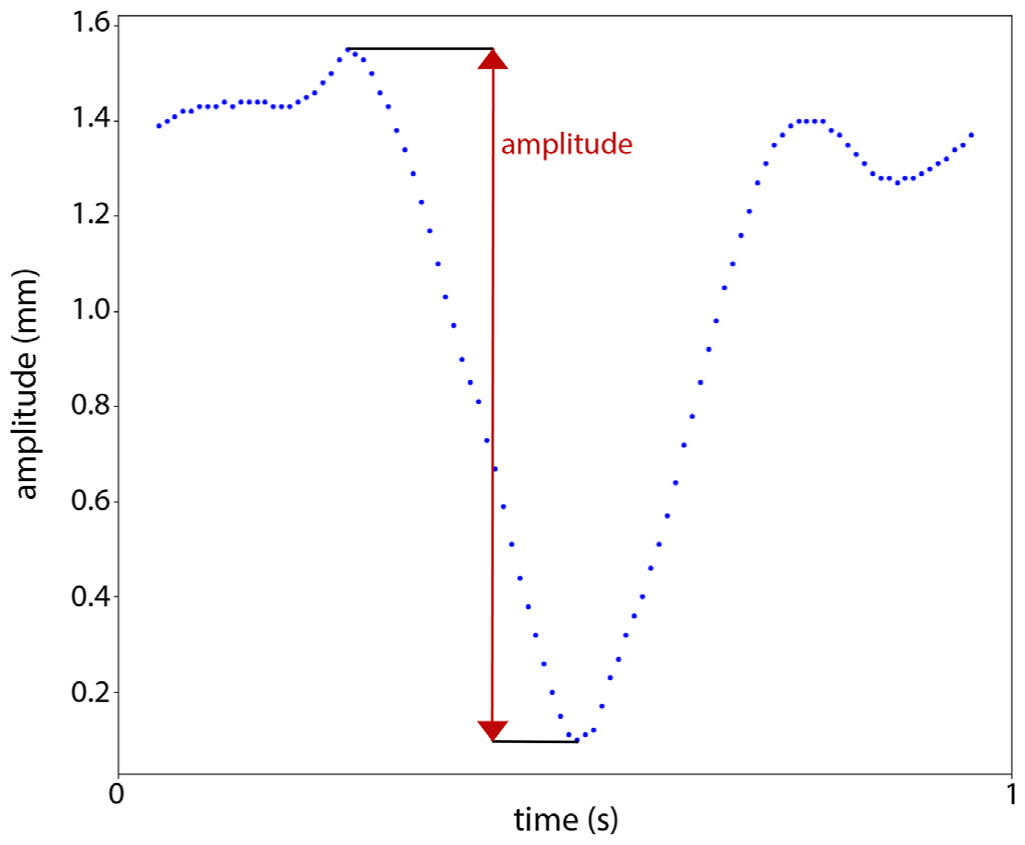
The shape of the pressure profile in the aortic artery used as input for the wave form generator adapted from Hazer et al.^14^ The amplitude of the motion pattern is shown in red.

The shape of a blood pressure profile in the aorta as reported by Hazer et al^14^ was programmed in the waveform generator (Fig 2). This wave form was used as input signal for the linear actuator to mimic the aortic motion. The baseline measurement was defined at a simulated heart rate of 70 beats per minute (BPM) and an absolute stroke amplitude of 1.5 mm, based on literature about the average heart rate^15^, ^16^, ^17^ and aortic motion.^1^, ^2^, ^3^ Because the experimental setup only produced motion in one direction, the baseline measurement was done only in the z-direction of the CT scanner. The baseline measurement was repeated two times for validation purposes. To study the influence of the heartrate, the frequency was changed from 50 to 90 BPM with intervals of 10 BPM. To study the influence of the amplitude, amplitudes of 1.0 and 2.0 mm were set. To study 3D motion the experimental setup was placed diagonally in the CT scanner with an elevation of one end of the linear actuator introducing 3D motion, that is, displacement not only in the z-direction (craniocaudal direction in clinical situations as depicted in Fig 1), but also in the *x* (lateral) and *y* (anterior-posterior) directions.

### Image acquisition

The ECG-gated CT angiography scans were acquired on a Somatom Definition Flash CT scanner (Siemens Healthineers, Erlangen, Germany) and on a Brilliance iCT 256 (Philips Healthcare, Eindhoven, the Netherlands) with standardized protocols, based on standard abdominal aortic aneurysm and EVAR follow-up CT scans. Scan parameters can be found in Table I. The raw data were retrospectively reconstructed into scans with 8 and 10 phases so that 8 or 10 equally sized phases of the cardiac cycle were obtained. The scans with eight phases were reconstructed at 0%, 13%, 25%, 37%, 50%, 63%, 75%, and 88% of the RR interval. The scans with 10 phases were reconstructed every 10% from 0% to 90% of the RR interval.

**Table I:** The computed tomography (CT) scan parameters for the two CT scanners

Scan parameter	Siemens Somatom Definition Flash	Philips Brilliance iCT 256
Tube voltage, kV	120	120
Tube current, mA	40	63 (52 to 65)^a^
Pitch	0.26 (0.18-0.34)^b^	0.18
Collimation, mm	2 × 64 × 0.600	128 × 0.625
Rotation time, s	0.285	0.27 (0.27-0.33)
Slice thickness, mm	1	1
Slice increment, mm	0.5	0.5
Reconstruction matrix	512 × 512 pixels (ca. 0.3 × 0.3 mm)	512 × 512 pixels (ca. 0.3 × 0.3 mm)
Reconstruction technique	iMAR	iDose

a Determined via automated tube modulation, presented as median (minimum-maximum).

b Determined automatically based on heart rate, presented as median (minimum-maximum).

### Image processing

**Fig 3:**
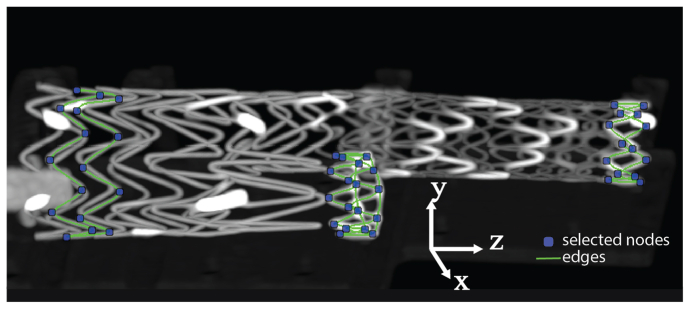
Maximal intensity projection of a phase-averaged computed tomography (CT) volume of the stent graft in the setup. The models resulting from the segmentation of the three individual stent rings are visualized as nodes (blue) and edges (green). The nodes were used to calculate the motion amplitudes and path lengths. The edges connect the nodes over the stent wire frame.

The motion calculations were performed on the ECG-gated CT data by applying image registration and stent segmentation algorithms in Python programming language (version 3) that have been previously explained and validated.^2^^,^^4^^,^^18^, ^19^, ^20^ The registration algorithm provides a phase-averaged CT volume with increased signal to noise ratio, and deformation fields that describe the displacement of each phase-averaged CT voxel back to the original cardiac phases. This process allows for performing only one measurement per scan that is automatically translated to the original phases, avoiding the need for repeated measurement in the different cardiac phases and thereby diminishing the user dependence of the measurements. The segmentation algorithm is used to obtain a geometric model of the wire frame of three selected stent rings in the phase-averaged CT volume. The models consist of nodes at the apices of the stent rings and edges connecting these nodes (Fig 3). The nodes were used to calculate the motion by translating the nodes' locations to their original locations in the individual phase CT volumes by backward-mapping of the deformation fields. Figures B3 and S.B5 of Appendix B shows examples of the motion amplitudes during the simulated cardiac cycle for the sampled sensor data, reconstructions into 8 phases and reconstructions into 10 phases for the two scanners.

Motion amplitudes were calculated for three directions: *x* (lateral), *y* (anterior-posterior), and *z* (craniocaudal) (Fig 1). The measured motion amplitudes were defined as the absolute displacement in one direction during a cardiac cycle, that is, the maximal displacement in that direction from two ends of the motion pattern as displayed in Fig 2. All measurements had their motion only in the *z* direction (craniocaudal motion in clinical situation as depicted in Fig 1), except for the 3D measurement. The 3D measurements also incorporated motion in the *x* (lateral) and *y* (anterior-posterior) directions. The sensor only measured the displacement in the single direction of the linear actuator. For all measurements, except the 3D measurements, this was the only direction of motion, so accurate comparisons could be performed. However, the 3D measurements also had motion in the *x* and *y* directions, so the sensor data were not accurate for the *x*-, *y*-, and *z*-motion amplitude measurements of the 3D measurements. Moreover, the 3D positions of the setup could not be reproduced accurately in the different scanners. Therefore, the 3D measurements were only compared between the reconstructions into 8 and 10 phases for amplitude measurements and not among scanners or to the sensor data. As a secondary measure, the traveled path length during a cardiac cycle was defined as the sum of the distances between the subsequent locations of a single node in the 8 or 10 phases during cycle, including from the final phase back to the first phase. All comparisons could be analyzed for the traveled path length, including the comparisons with the sensor and among the scanners for the 3D measurements.

### Statistical analysis

Continuous data were normally distributed based on visual inspection and are presented as mean ± standard deviation (minimum-maximum). The calculated motion of the different reconstructions (sensor data and reconstructions into 8 phases and 10 phases on Siemens and Philips scanners) was compared by use of a one-way analysis of variance for repeated measures, with Bonferroni post hoc testing. The difference in motion between reconstruction into 8 and 10 phases for the 3D measurements were compared using paired samples t-tests. Differences in the 3D motion between the Siemens and Philips scanner were not calculated because the position of the setup in the scanner could not be reproduced accurately. Because the sensor only measured motion in one direction only the path lengths, and not the *x*-, *y*-, and *z*-amplitudes of the 3D measurements, could be compared to the sensor. Test results were presented as mean difference with the 95% confidence interval (CI). Statistical significance was assumed when the *P* value was <.05. Statistical analyses were performed using IBM SPSS Statistics for Windows (version 25.0; IBM Corporation, Armonk, NY).

## Results

**Fig 4:**
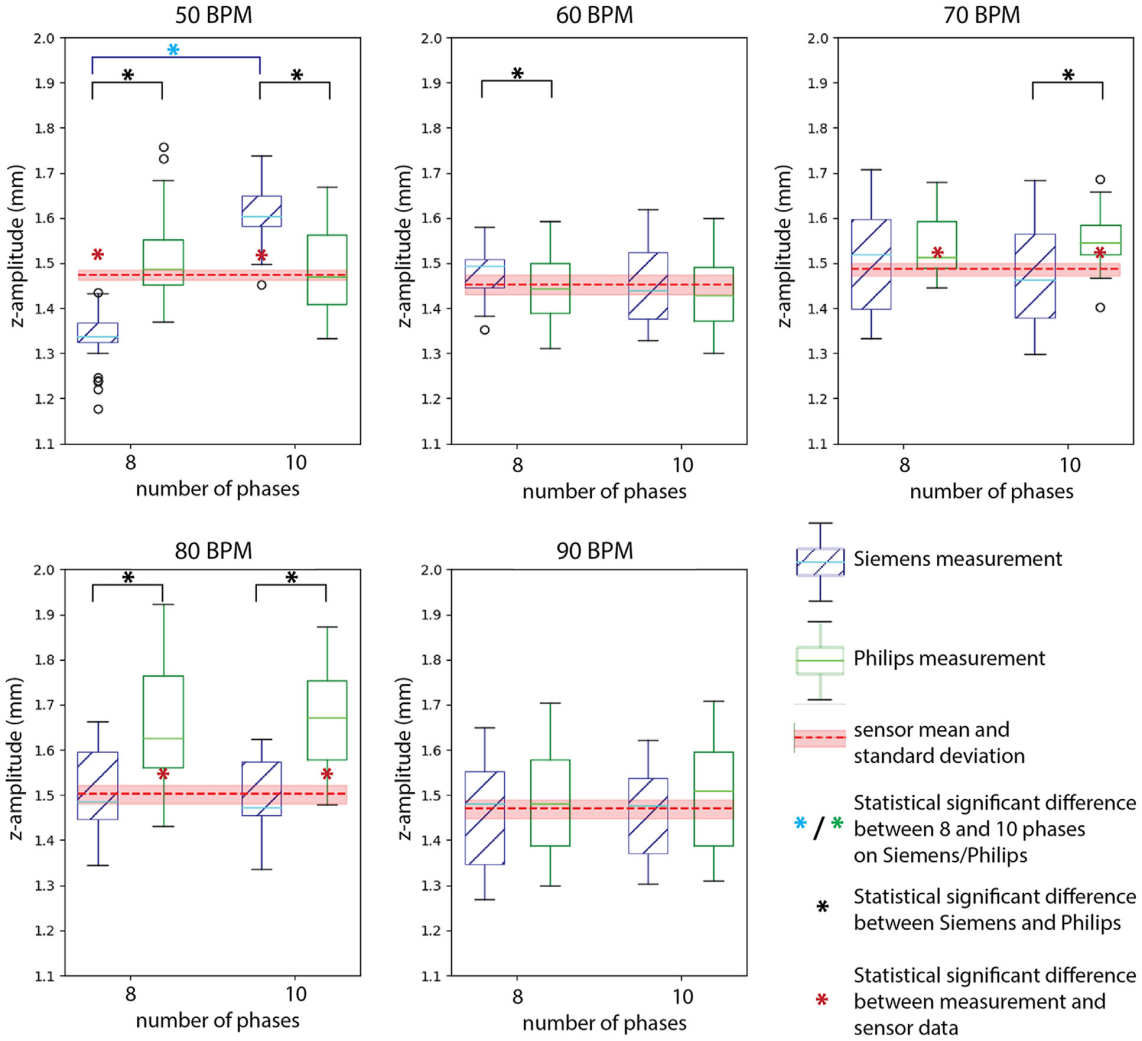
Boxplots of the z-amplitude motion measurements for the Siemens (blue) and Philips (green) scans, reconstructed to either 8 or 10 cardiac phases, at different cardiac frequencies: 50- 90 beats per minute (*BPM*). The mean (dashed red line) and standard deviation (light red band) are shown for each sensor measurement as well.

**Fig 5:**
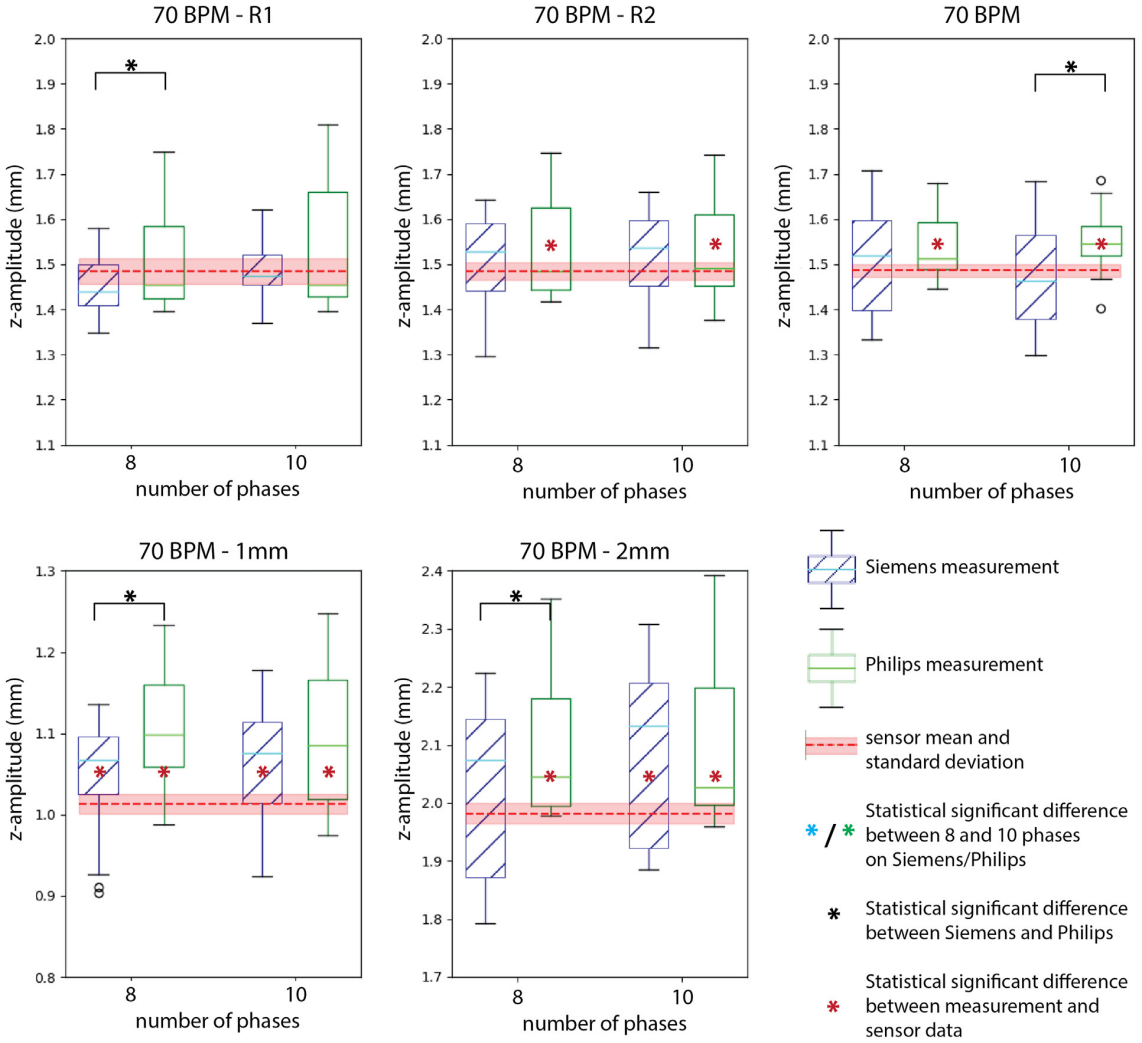
Boxplots of the z-amplitude motion measurements for the Siemens (blue) and Philips (green) scans, reconstructed to either 8 or 10 cardiac phases, for variations to the baseline measurement at 70 beats per minute (*BPM*) with a motion amplitude of 1.5 mm: two repeated measurements (R1 and R2) and amplitude variation (1.0 mm and 2.0 mm). The mean (dashed red line) and standard deviation (light red band) are shown for each sensor measurement as well.

The rings segmentations resulted in a total of 48 nodes per measurement that were used to calculate the potential differences in motion quantification. Overall, the difference between the CT measurements and the sensor data was 0.04 mm (95% CI, 0.03-0.04 mm; *P* = .002) for the z-amplitudes and 0.14 mm (95% CI, 0.13-0.15 mm; *P* < .001) for the path lengths (Figs 4 and 5 and Appendix C). The repeated measurements of z-amplitude and path length at baseline settings (70 BPM motion with a 1.5 mm amplitude) did not differ with respect to the original baseline measurement (Appendix C). The complete results of the statistical analyses can be found in (Appendix C).

### Reconstructions into 8 vs 10 phases

The z-amplitudes did not show a significant difference between the reconstructions into 8 and 10 phases when combining all measurements (Table II). When comparing the individual z-amplitude measurements shown in Figs 4 and 5, only at a simulated heart rate of 50 BPM on the Siemens scanner the reconstructions into 8 and 10 phases showed a statistical significant difference of 0.27 mm (95% CI, 0.23-0.31 mm; *P* < .001).

**Table II:** The differences between the 8 and 10 phases computed tomography (CT) reconstruction of all calculated amplitudes in *z* direction (craniocaudal direction, z-amplitude), *y* (anterior-posterior direction, y-amplitude^a^), and *x* (lateral, x-amplitude^a^), and path lengths

Characteristics	Siemens Somatom Definition Flash	Philips Brilliance iCT 256	Both scanners combined
z-Amplitude, mm	0.04 (−0.01 to 0.09, *P* = .564)	0.00 (−0.05 to 0.05, *P* = 1.000)	0.02 (−0.01 to 0.05, *P* = .358)
Path lengths, mm	0.08 (0.03 to −0.18, *P* = .440)	0.07 (−0.04 to 0.17, *P* = .889)	0.07 (0.01 to 0.13, ***P* = .013**)
3D^a^			
x-Amplitude, mm	0.07 (0.06 to 0.09, ***P* < .001**)	0.01 (−0.01 to 0.03, *P* = .225)	0.04 (0.03 to 0.05, ***P* < .001**)
y-Amplitude, mm	0.05 (0.04 to 0.06, ***P*<.001**)	−0.01 (−0.01 to −0.00, ***P* = .044**)	0.02 (0.01 to 0.03, ***P* < .001**)
z-Amplitude, mm	0.16 (0.15 to 0.17, ***P* < .001**)	−0.05 (−0.06 to −0.03, ***P* < .001**)	0.06 (0.03 to 0.08, ***P* < .001**)
Path lengths, mm	0.51 (0.43 to 0.58, ***P* < .001**)	−0.04 (−0.07 to −0.02, ***P* < .001**)	0.23 (0.17 to 0.29, ***P* < .001**)

The continuous data are presented as mean (95% confidence interval, *P* value).

Boldface entries indicate statistical significance.

a The 3-dimensional measurements, in which the setup was placed diagonally in the scanner, are presented separately from the other measurements.

The calculated path lengths seemed to differ between the reconstructions into 8 and 10 phases when all combined into 1 analysis, but this effect is not seen when the path lengths are analyzed per scanner (Table II). When the individual measurements were compared, path lengths differed between reconstructions into 8 and 10 phases for the Siemens 50, 60, and 70 bpm-2 mm measurements and the Philips 70 bpm measurements (Appendix C). Still, these differences were <0.50 mm, which is the slice thickness of the CT scans.

The 3D measurements showed statistical significant differences in x-, y-, and z-amplitudes between reconstructions into 8 and 10 phases of up to 0.2 mm, except for the *x* directions (lateral motion) of the Philips scanner (*P* = .225) (Table II). This table also shows that the difference in 3D path lengths between the reconstructions into 8 and 10 phases is larger for the Siemens scanner ((≤0.6 mm) than for the Philips scanner (≤0.1 mm).

### Siemens vs Philips scanner

Table III presents the comparison of the scanners that resulted in a difference in z-amplitude for all measurements combined of 0.04 mm (95% CI, 0.01-0.07 mm; *P* = .003). However, for the reconstructions into 10 phases, no significant difference was established. For the individual measurements, the z-amplitude differed between the CT scanners in 6 reconstructions into 8 phases and 4 reconstructions into 10 phases (Figs 4 and 5). Still, these difference were all well below 0.3 mm, which is the spatial resolution of the CT scans.

**Table III:** The differences between the Siemens Somatom Definition Flash and Philips Brilliance iCT 256 CT scanners in the calculated amplitudes in *z*-direction (craniocaudal direction, z-amplitude) and path lengths

Characteristics	Eight phases reconstructions	Ten phases reconstructions	Eight and 10 phases combined
z-Amplitude, mm	0.06 (0.01 to 0.11, ***P* = .009**)	0.03 (−0.02 to 0.08, *P* = 1.000)	0.04 (0.01 to 0.07, ***P* = .003**)
Path lengths, mm	0.13 (−0.03 to 0.24, ***P* = .003**)	0.12 (0.02 to 0.23, ***P* = .010**)	0.13 (0.06 to 0.17, ***P* < .001**)
3D^a^ path lengths, mm	0.16 (0.13 to 0.18, ***P* < .001**)	−0.39 (−0.41 to −0.37, ***P* < .001**)	−0.12 (−0.18 to −0.06, ***P* < .001**)

The continuous data are presented as mean (95% confidence interval, *P* value).

Boldface entries indicate statistical significance.

a The three-dimensional (3D) measurements, in which the setup was placed diagonally in the scanner, are presented separately from the other measurements.

Scanner differences were also observed in the path lengths calculations for nearly all measurements (Appendix C), with an overall difference of 0.13 mm (95% CI, 0.06-0.17 mm; *P* < .001) (Table III). The difference between the scanners in path lengths of the 3D measurements was 0.12 mm (95% CI, 0.06-0.18 mm; *P* < .001). This difference was greater for the reconstructions into 10 phases than for the reconstructions into 8 phases (Table III).

## Discussion

This study evaluated the comparability of motion calculation on reconstructions into 8 and 10 phases of ECG-gated CT scans on two CT scanners from different vendors. The CT scans were obtained for an experimental setup with a stent graft moving according to the aortic pressure wave. These in vitro results demonstrated that motion amplitudes can be accurately assessed without any clinically significant difference between reconstructions into 8 and 10 phases, or between the used CT scanners. The overall observed differences in motion amplitudes were well within the size of a voxel of the phase-averaged CT volume of 0.3 × 0.3 × 0.5 mm. The calculated total traveled path length during one cardiac cycle differed up to maximally 0.6 mm between the different reconstructions and scanners. Preferably, the CT parameters should be similar among CT scans that are being compared in clinical trials and clinical situations, as was the case in the present work. These parameters include tube voltage, collimation, slice thickness, slice increment, and reconstruction matrix. Note that the smaller the slice thickness and slice increment, the more accurate the motion quantification may be performed, because the accuracy of motion quantification is limited by the voxel size, particularly the slice thickness of the CT volumes.

The dynamic behavior of stent grafts in the aorta has been reported in literature,^3^^,^^4^^,^^9^^,^^13^^,^^21^, ^22^, ^23^, ^24^ including papers that have implemented the same image registration and segmentation techniques as applied in the present study.^3^^,^^4^^,^^13^^,^^21^^,^^22^ Validation of these algorithms has been performed by Koenrades et al,^2^ including accuracy assessment for motion calculation in ECG-gated CT scans with reconstructions into 10 phases on CT scanners from two vendors (Somatom Definition Flash as used in the current study and the Aquilion 64, Toshiba Medical Systems Corporation, Otawara City, Tochigi Prefecture, Japan). For this validation, they used an experimental setup and found an absolute error of at most 0.3 mm for motion calculation, with average errors of 0.12 and 0.14 mm for the respective scanners. These results are comparable with the results that we found in the present study. Yet, they only evaluated the motion pattern in z-direction, whereas the present study also included comparison of the total traveled path lengths and a 3D measurement. Furthermore, Koenrades et al only evaluated reconstructions into 10 phases of simplified motion patterns, although reconstructions into 8 phases are also common. Theoretically, quantifying cardiac-pulsatility-induced motion with 8 or 10 phases in an ECG-gated CT angiography might be an undersampling of the motion signal, especially for reconstructions into 8 phases. For example, Simmering et al^13^ investigated the postoperative behavior of 2 different iliac branched devices, scanned with different scanning protocols: reconstruction into 8 phases or 10 phases on a Philips Brilliance iCT 256 scanner. Initially, there may have been doubt as to whether the observed difference between the stent grafts was caused by the stent graft type or the scan type. However, the present work revealed that the scan type does not significantly influence the motion quantification and the observed differences could indeed be attributed to the different stent grafts. This was also further substantiated in Appendix B, where it can be seen that there is good visual similarity between the different measurements. Hence, ECG-gated CT scans may be reconstructed into eight cardiac phases that could increase the clinical applicability of these types of CT scans. However, when technological improvements of CT scans and scanners improve in spatial resolution, the accuracy of the motion quantification should be reevaluated.

It is important for the motion analysis to document the patient's heart rate during scan acquisition. The measurements at a cardiac frequency of 50 BPM on the Siemens scanner revealed an underestimation of circa 0.14 mm of the z-amplitude when the data are reconstructed into 8 cardiac phases and an overestimation of circa 0.13 mm when reconstructed into 10 cardiac phases, leading to an average difference in motion amplitude of 0.27 mm between the reconstructions into 8 and 10 phases. The theoretical justification of the number of phases in the ECG-gated CT scans for accurate motion quantification in Appendix B could not provide an explanation for the relatively large difference in motion amplitude on the 50-BPM measurement. An explanation for this observation was sought in the acquisition of the scan. The literature shows that the combination of scan parameters (pitch and rotation time) and heart rate should be in balance to ensure accurate motion quantification.^6^ The minimum heart rate can be calculated with the following equation^6^:

$$minimum\;heart\;rate=\frac{60\times pitch}{T_{rotation}}$$

with a pitch of 0.18 and a rotation time of 0.285 second, indicating a minimum heart rate of $\frac{60\cdot 0.18}{0.285}=38$ BPM for the Siemens scanner. This also does not provide an explanation for the difference in amplitude calculation. Nevertheless, caution is advised when evaluating motion calculations on ECG-gated CT in patients with a low heart rate, because it may induce an inaccurate motion calculation.

Even though the difference in amplitude measurement between the scanners is smaller than the voxel size, it is important to consider the difference in reconstruction techniques used to create the CT images. The Siemens scanner used the iMAR reconstruction technique, which is an iterative CT reconstruction technique specifically developed to decrease metal artifacts.^25^^,^^26^ The Philips scanner used the iDose^3^ reconstruction technique, which is a hybrid iterative reconstruction algorithm that allows for radiation dose reduction as lower noise, including metal artifacts that may arise in stent graft imaging.^27^^,^^28^ However, the metal stents appeared brighter and with some blooming artifact on the Philips scans compared with the Siemens scans, suggesting the artifact noise suppression of iDose^3^ might be inferior to that of the iMAR technique in stent graft imaging. Even though this appears not to lead to clinically significant differences or errors of the measurements, it might play a part in the z-amplitude and path length measurements on both the reconstructions into 8 and 10 phases being overestimated on the 80-BPM Philips measurement by 0.16 and 0.18 mm, because no other explanation was found for this observation. In addition, it is important to consider the findings of Velu et al,^29^ who compared different CT scanners for anatomical measurements. They revealed that the difference in anatomical measurements may on different CT scanners can exceed the clinical tolerable margin of 1 mm albeit this was not observed in this study.

### Limitations

This experimental setup assumed regular heart rates. However, in clinical situations patients could have an arrythmia. To what extend that may influence the image reconstructions, and consequently the motion calculations, should be further investigated. Furthermore, the accuracy of the motion quantification appears to be limited by the resolution of the CT scan. This finding indicates that future research should focus on improving scan resolution without increasing, or rather decreasing, the radiation exposure for the patients. The experimental design used in this work did not take into account resistance of any kind, which may be considered a limitation. However, the authors assumed that the air in which this setup was placed did not produce any resistance to the stent graft motion produced by the linear actuator. This setup solely focused on the quantification of displacement of a stent graft and how accurately this could be captured using ECG-gated CT. Because this work showed that retrospective ECG-gated CT scans can accurately capture the displacement of a stent graft, we can assume that they can also capture the displacement of in vivo stent graft, even when this displacement is influenced by the resistance of surrounding structures.

## Conclusions

Using an experimental setup simulating the stent graft motion in the aorta according to a blood pressure wave, the comparability of motion calculation on retrospective ECG-gated CT scans reconstructed into 8 and 10 phases made with two CT scanners from different vendors were evaluated. The following conclusions could be drawn:

• The simulated cardiac-pulsatility motion could be accurately assessed without clinically significant differences between reconstructions into 8 and 10 phases, or between used Siemens and Philips CT scanners, and with good repeatability.
• Motion analysis results of clinical and research investigations obtained using different types of ECG-gated CT scans and CT scanners can be compared up to the accuracy of the CT scan's resolution.
• Retrospective ECG-gated CT scans, made either in the context of clinical trials or in the context of clinical workflow, can be accurately compared even when the scans are not made with the same scan protocol nor scanner type. This in turn allows for wider application of this type of CT scans in clinical and research settings that eventually provides more insights than the current standard of static CT.

## Author Contributions

Conception and design: JS, DZ, RG, HK, GS, MR, CS

Analysis and interpretation: JS, DZ, RG, MR, CS

Data collection: JS, DZ

Writing the article: JS

Critical revision of the article: JS, DZ, RG, HK, GS, MR, CS

Final approval of the article: JS, DZ, RG, HK, GS, MR, CS

Statistical analysis: JS

Obtained funding: Not applicable

Overall responsibility: JS

## Disclosures

M.R. is a consultant for W. L. Gore & Associates.
