# Supplementary material for: The influence of electrocardiogram-gated computed tomography reconstruction into 8 or 10 cardiac phases on cardiac-pulsatility-induced motion quantification of stent grafts in the aorta
Source: JVS Vasc Sci. 2023 Sep 28;4:100131. doi: 10.1016/j.jvssci.2023.100131 (PMC10682660; doi:10.1016/j.jvssci.2023.100131)
Supplement: Appendix A [file mmc1.pdf]

Supplemental Material A – The Experimental Set-up

The complete experimental set-up is schematically depicted in Figure S.A1. The linear and how it is stimulated by the waveform generator that in turn is triggered by the ECG-simulator was described in the main text. The trigger needs amplification to reach the input threshold of the waveform generator. This is achieved by a custom-made comparator circuit with an operational amplifier of which the schematics are shown in Figure S.A2. The linear actuator is powered by two power supplies (EA-PS 512-21-T, Netzgerät Power Supply, EA Elektro-Automatik). The Hall sensor value is processed by an Arduino Microcontroller (Pro Micro 5V 16M Mini Leonardo Arduino Microcontroller) at a speed of 100 samples per second that is programmed with specialized Arduino code (Supplemental Material B2). This sensor data was recorded on a laptop using the Processing code (Processing 3, see Supplemental Material B3).

The Hall sensor is mounted to the lever of the linear actuator and located between two magnets at the base of the linear actuator. The changing magnetic field experienced by the sensor as it moves between these magnets produces the output of the sensor. The Microcontroller required calibration which was achieved manually using a M3 screw (1 revolution induces a 0.5 mm displacement of the screw's end). To initiate the calibration, the maximal stroke of -3.00 mm is set and the corresponding sensor data-value is noted. The screw is rotated 180° so the stroke is -2.75 mm and the corresponding sensor data-value is noted. This process is repeated until a stroke of +3.00 mm is reached. The resulting sensor data-values are then used as input for "sensorval" in the Arduino code.

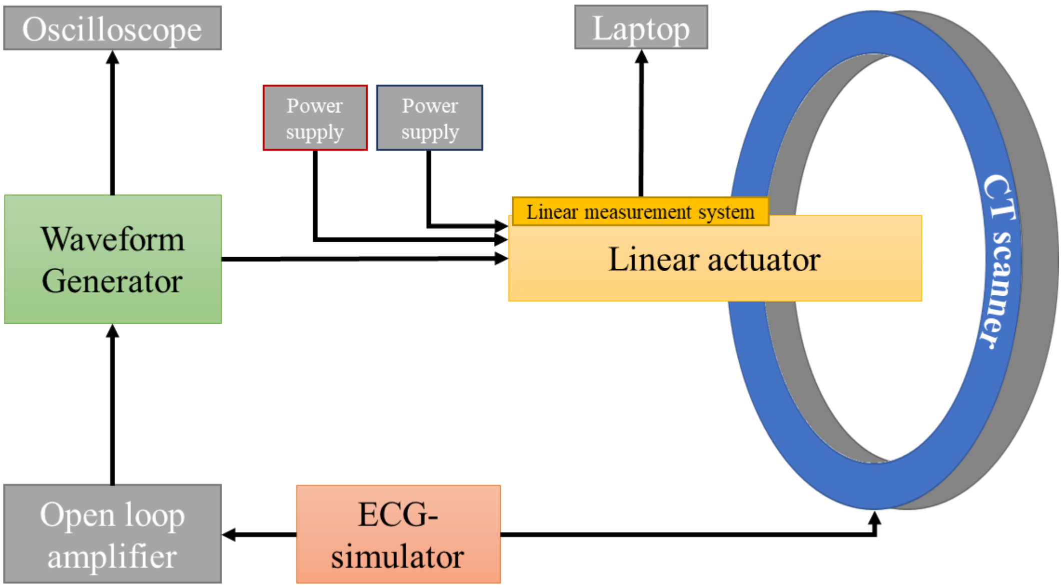

***Figure S.A1** – Schematic representation of the experimental set-up*

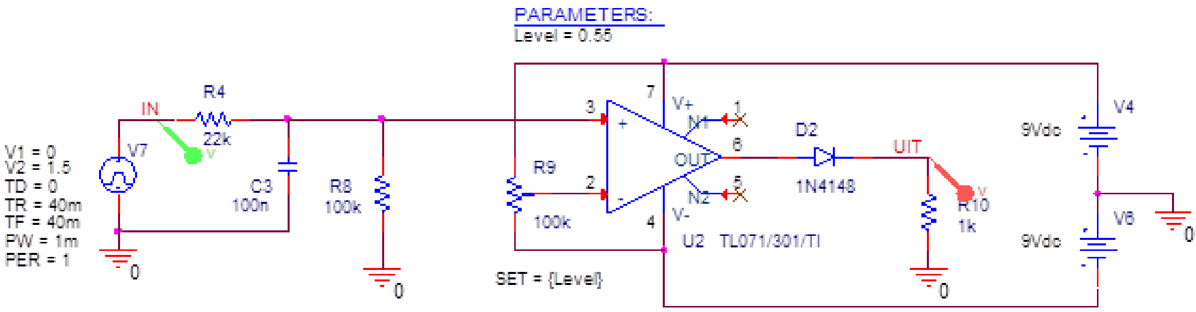

***Figure S.A2** – Schematic representation of the comparator circuit with an operational amplifier.*

Supplemental Material A2 – Arduino Microcontroller Script

analogRead_OneHall-sensor_ProMicro

```
/* Example for analogRead on Pro Micro (32U4, 3V3)
*/
#define LED_BUILTIN 17
#define ADC0_pin A3

const uint16_t  sensorval[] = {115,140,192,241,285,327,360,396,428,461,490,523,555,586,614,650,681,722,759,803,824};
uint16_t    ADC0_value;
uint16_t    ZeroValue = 0;
uint16_t    n_10ms = 1;
float       Position = 0.0;
uint32_t    now_time, prev_time, elapsed_time, next_sample, period = micros();

//=====================
void setup() {
//=====================

    pinMode(LED_BUILTIN, OUTPUT);
    pinMode(ADC0_pin, INPUT);
    Serial.begin(115200);
    delay(1000); // relax, let the serial port be configured.

    ZeroValue = analogRead(ADC0_pin); // read the "zero position" value

    delay(100);
    period = 9990;
    next_sample = micros() + period;
}

void loop() {

  String text = "";

    // wait
    while ( next_sample > micros() ) {} // wait till next sample moment
    now_time = micros();
    next_sample = now_time + period;
    elapsed_time = now_time - prev_time;
    prev_time = now_time;
    n_10ms += 1;

    // Single read
    ADC0_value = analogRead(ADC0_pin); // read a new value
//    Serial.println(ADC0_value);

    int i=0;
    while ( sensorval[i] <= ADC0_value) { i++; }
    i--;
    Position = 0.25 / (sensorval[i+1] - sensorval[i]) * (ADC0_value - sensorval[i]);
    Position += -3.0 + 0.25 * (i);

    text = " ";
    Serial.print(ADC0_value + text + Position + "\n");

    // for calibration and performance check
/*    n += 1;
    text = " "; // define string to start concatenation
    text = text + "[" + elapsed_time + "] ";
    text = text + ADC0_value;
    text = text + text + n;
    Serial.print(text + "\n");
*/
    if (n_10ms > 50) {
      n_10ms = 1;
      digitalWrite(LED_BUILTIN, !digitalRead(LED_BUILTIN));
    }


} // loop
```

Supplemental Material A3 – Processing Script

ECGgatedCT_experiment

```
// Gerben te Riet Scholten
// 2021-03-15

import processing.serial.*;
Serial myPort;
Table myTable;
String filename;
String feature = "stil";
PrintWriter outputFile;
int lf = 10;    // Linefeed in ASCII

void setup() {
  printArray(Serial.list());        // List all the available serial ports
  myPort = new Serial(this, Serial.list()[3], 115200);

  filename = "Data/DEBORAH";
  filename = filename + "-" + str(year()) + "-" + str(month()) + "-" + str(day());
  filename = filename + "_" + str(hour()) + "h" + str(minute()) + "m" + str(second()) + "s";
  filename = filename + "_" + feature;
  filename = filename + ".txt";
  outputFile = createWriter(filename);

  size(400,600);
  background(145,25,225);
}

void draw() {

  while (myPort.available() > 0) {
    String inBuffer = myPort.readStringUntil(lf);
    if (inBuffer != null) {
      print (inBuffer);
      outputFile.print(inBuffer);
//      outputFile.println(inBuffer.length());
    } //if
  } //while
} //draw

void keyPressed()
{
  outputFile.flush();  // Writes the remaining data to the file
  outputFile.close();  // Finishes the file
  println("Stopped, now listing data:");
    String[] lines = loadStrings(filename);
    println("there are " + lines.length + " lines");
    for (int i = 0 ; i < lines.length; i++) {
      println(lines[i]);
    }
  exit();
}
```
