# Supplementary material for: The influence of electrocardiogram-gated computed tomography reconstruction into 8 or 10 cardiac phases on cardiac-pulsatility-induced motion quantification of stent grafts in the aorta
Source: JVS Vasc Sci. 2023 Sep 28;4:100131. doi: 10.1016/j.jvssci.2023.100131 (PMC10682660; doi:10.1016/j.jvssci.2023.100131)
Supplement: Appendix B [file mmc2.docx]

**Supplemental material B – Theoretical comparison of 8 and 10 phases reconstructions.**

Theoretically, the cardiac pulsatility induced motion of *in situ* stentgrafts may be under sampled in ECG-gated CT scans, as only 8 or 10 samples are taken of the motion signal in terms of 8 or 10 phases of the scans, respectively. From that perspective, under sampling would be an even larger potential problem in 8 phases reconstructions (i.e. 8 samples of the signal) than in 10 reconstructions (i.e. 10 samples of the signal). To determine the minimal sample frequency of the motion signal, we assume the minimum-sampling rate at which a continuous-time signal can be uniformly sampled with complete recovery or reconstruction of the original signal:

$$f_{s}>2$$

In which $f_{s}$ is the minimal sample frequency and $W$is the Nyquist frequency, i.e. the highest frequency in the signal. Note however that the sampling is not momentarily, but is performed during the duration of a single cardiac phase over multiple cardiac cycles.

For example, a signal of 60 bpm indicates a cardiac frequency of 1Hz. However, this is not the frequency of the signal, because a 1Hz signal would be a pure sinus wave per second. Assuming the used blood pressure wave that is implemented at several heart rates, these heart rates are not the signal-frequencies, since the blood pressure wave is not a pure sinus. Hence, the blood pressure wave holds multiple frequencies. To determine the number of frequencies in the blood pressure wave a Fourier transformation is required. The induced motion of the experimental set-up (Figure S.C1) is measured with a sensor at sample frequency $f_{s}$ = 100 Hz. This signal is converted to a Power Spectrum (Figure S.C2) by computation of the one-dimensional discrete Fourier Transform using the function *fft* (as |fft|^2^ with windowing according to Welch) of the Python package NumPy showing the different frequencies in the 60 bpm blood pressure wave signal. Three distinct frequencies (1, 2, and 3 Hz) can be distinguished from this figure.


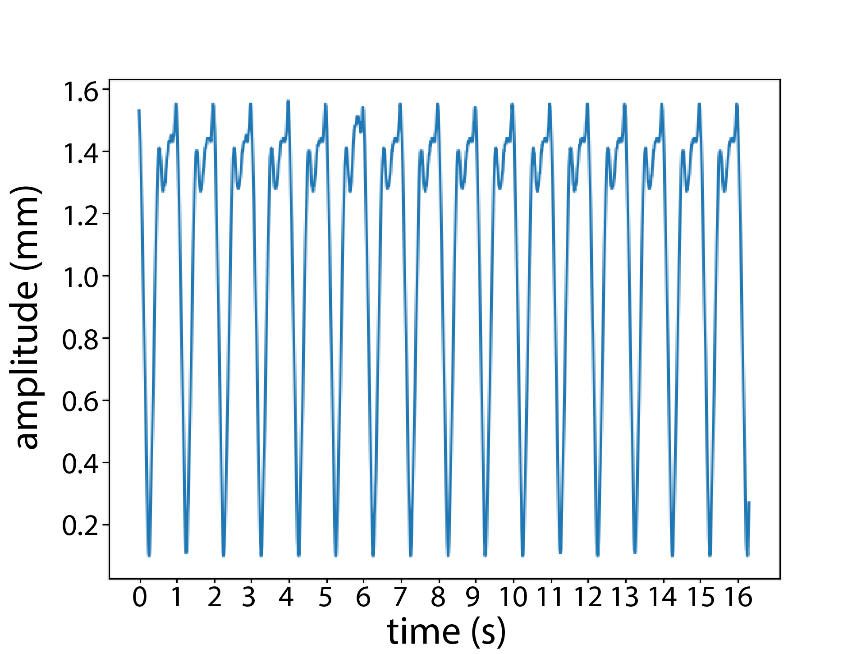


***Figure S.B1 –*** *Sensor data of the stentgraft motion according to the input signal (blood pressure wave) by Hazer et al.^14^ at a cardiac frequency of 60 bpm.*

Assuming the 3 Hz peak indicates the Nyquist frequency, the minimal sample frequency is 2×3 = 6 Hz, i.e. 6 samples per second, which is in this case 6 samples per heart beat since we use the example of a 60 bpm heart rate (1 heart beat per second). In this example, sampling the blood pressure wave with 8 or 10 phases would be sufficient to determine the motion of the stent graft. That this is the case can also be seen in Figure S.C3 that depicts the sensor data at 8 and 10 samples and the CT motion amplitudes in z-direction for the 60 bpm measurement on the Philips scanner. The sensor and CT measurements show similar patterns for the 8 and 10 samples/reconstructions.

When taking the minimal and maximal heart frequencies in the present study of 50 bpm and 90 bpm, respectively, the resulting sensor signals and power spectrums are as depicted in Figure S.C4. Both measurements show 3 main frequency peaks with the third at 2.5 Hz and 4.5 Hz for 50 bpm and 90 bpm, respectively. This would indicate that 8 phases reconstructions may induce some under sampling in 90 bpm measurements. However, the depiction of the sensor and CT motion measurements for the 8 and 10 samples/reconstructions in Figure S.C.5 reveals that this influence is not clinically relevant as was also revealed in the main documents results. This may relate to the sampling being performed during the duration of a single cardiac phase over multiple cardiac cycles and not at a single timepoint.


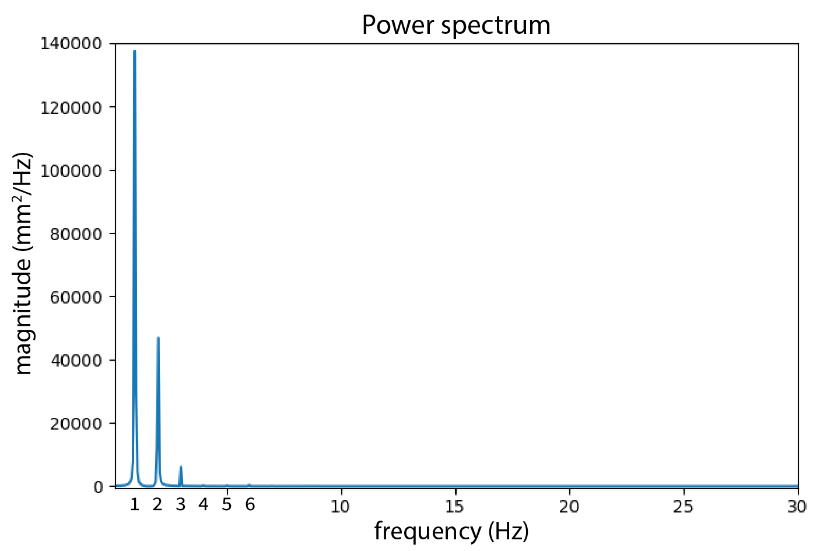


***Figure S.B2 –*** *The power spectrum of the sensor data of the stentgraft motion according to the blood pressure wave at a cardiac frequency of 60 bpm.*


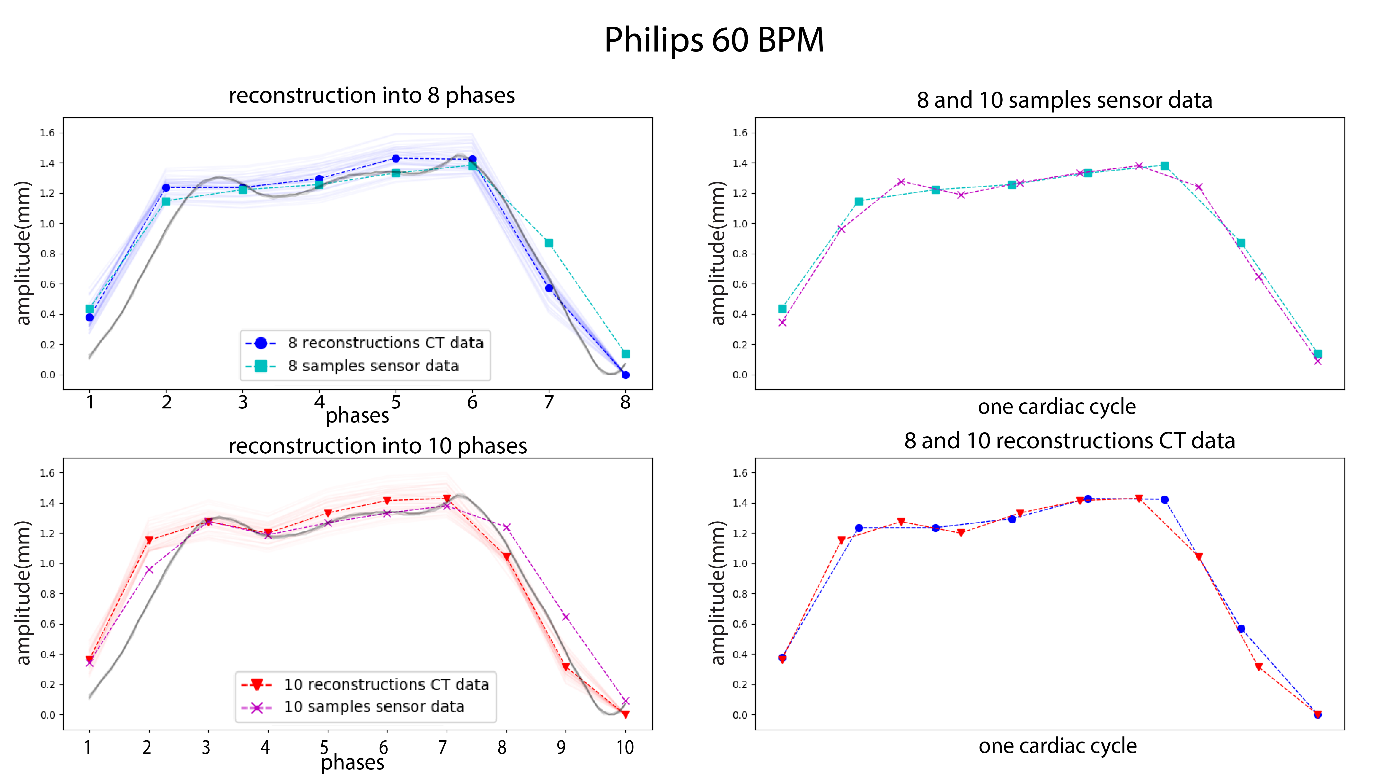


***Figure S.B3*** ***–*** *The mean computed tomography (CT) and sensor motion per cardiac phase for the 8 phases reconstruction (top left) and of the 10 phases reconstructions/samples (bottom left) of the 60 bpm measurement on the Philips CT scanner. The CT measured data is shown per node of the stent-ring segmentations in opaque blue and red for the 8 and 10 phases reconstructions respectively. The true sensor data is shown in grey. The 8 and 10 samples sensor motion data depicted together (top right) as well as the 8 and 10 phases reconstruction motion (bottom right) per phase.*


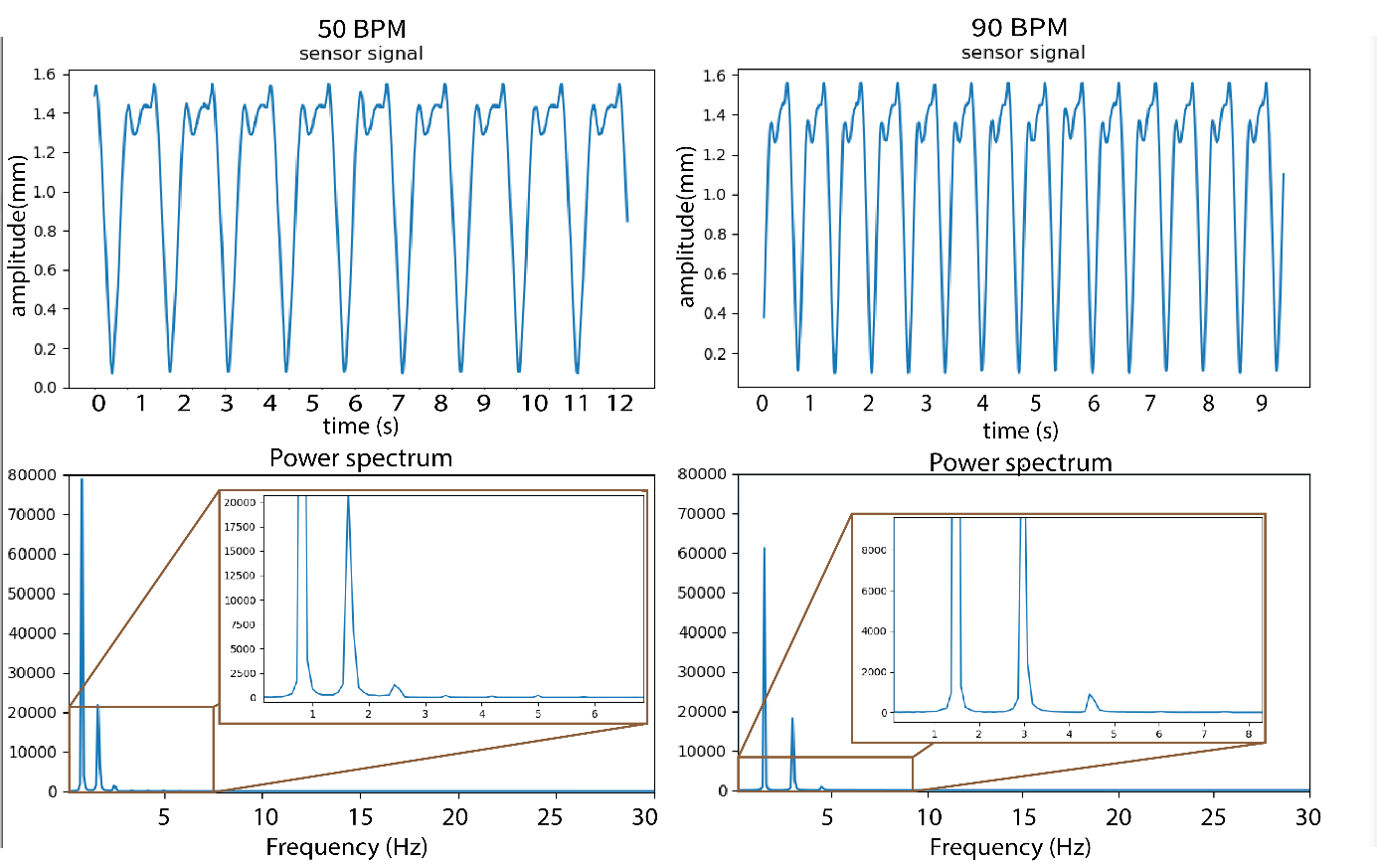


***Figure S.B4 –*** *The sensor signal of the 50 bpm and 90 bpm sensor data (top left and right, respectively) and the corresponding Power Spectra (bottom left and right, respectively).*


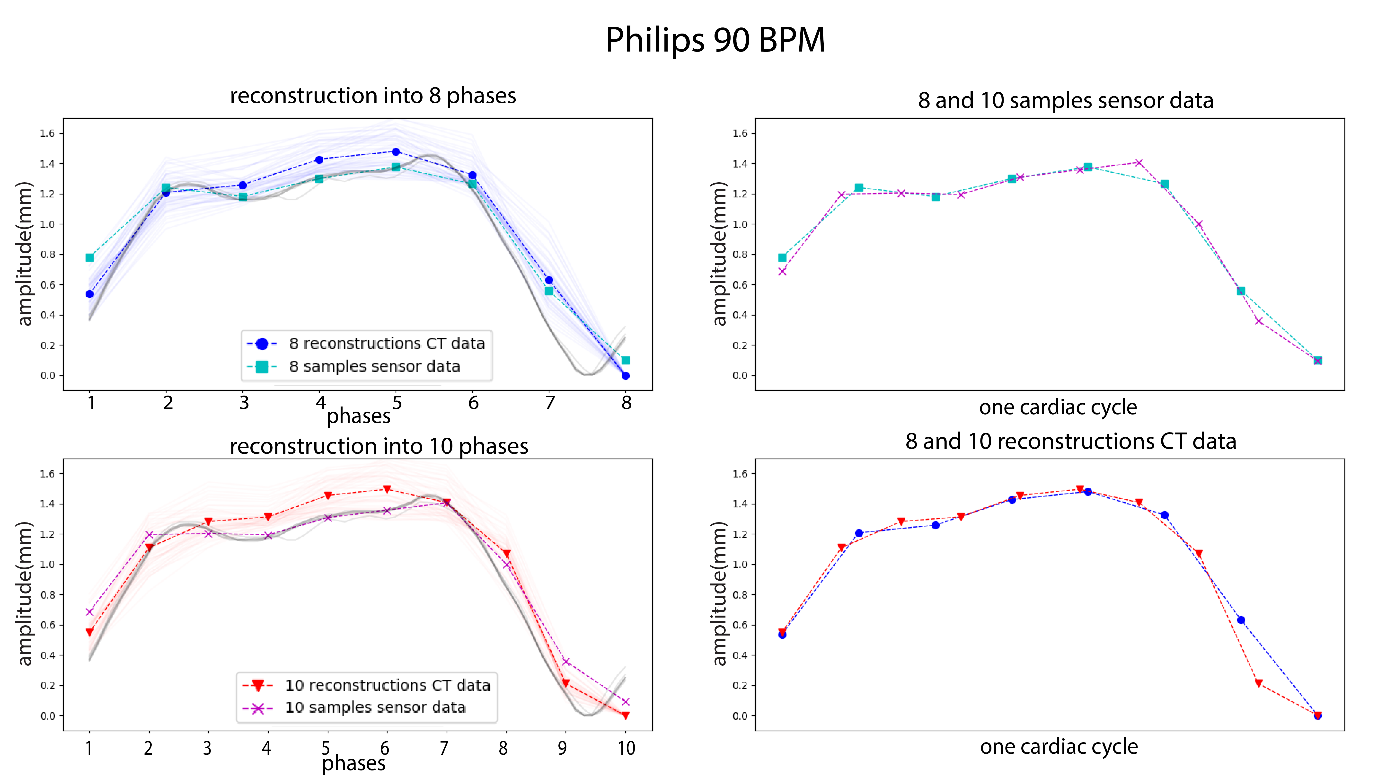


***Figure S.B5 –*** *The mean computed tomography (CT) and sensor motion per cardiac phase for the 8 phases reconstruction (top left) and of the 10 phases reconstructions/samples (bottom left) of the 90 bpm measurement on the Philips CT scanner. The CT measured data is shown per node of the stent-ring segmentations in opaque blue and red for the 8 and 10 phases reconstructions respectively. The true sensor data is shown in grey. The 8 and 10 samples sensor motion data depicted together (top right) as well as the 8 and 10 phases reconstruction motion (bottom right) per phase.*
