# Supplementary material for: The influence of electrocardiogram-gated computed tomography reconstruction into 8 or 10 cardiac phases on cardiac-pulsatility-induced motion quantification of stent grafts in the aorta
Source: JVS Vasc Sci. 2023 Sep 28;4:100131. doi: 10.1016/j.jvssci.2023.100131 (PMC10682660; doi:10.1016/j.jvssci.2023.100131)
Supplement: Appendix C [file mmc3.doc]

**Supplementary materials C – Comparisons between individual measurements**


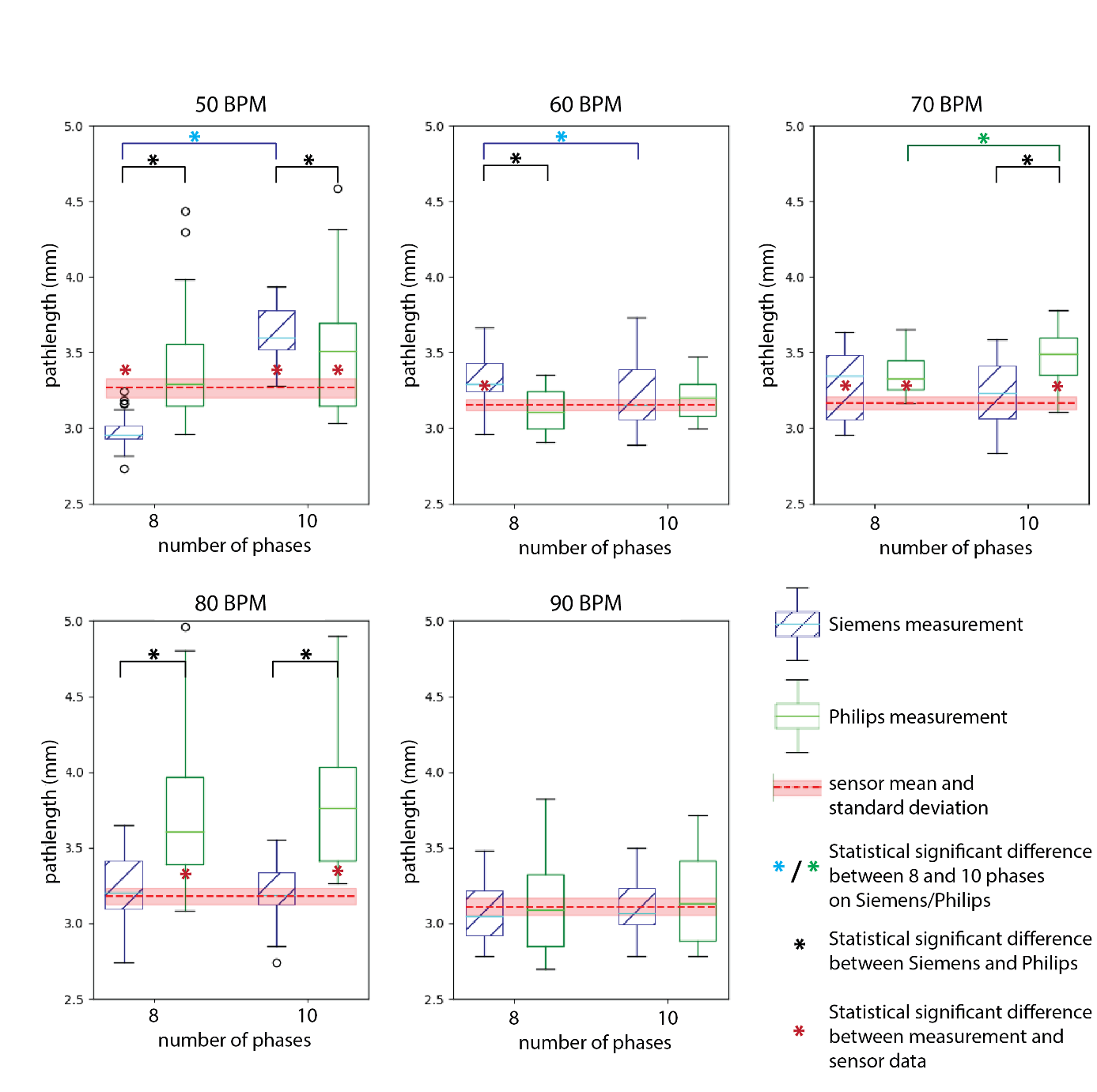
 ***Figure S.C1 –*** *Boxplots of the traveled pathlength measurements for the Siemens (blue) and Philips (green) scans, reconstructed to either 8 or 10 cardiac phases, at different cardiac frequencies: 50 – 90 beats per minute (BPM). The mean (dashed red line) and standard deviation (light red band) are shown for each measurement as well.*


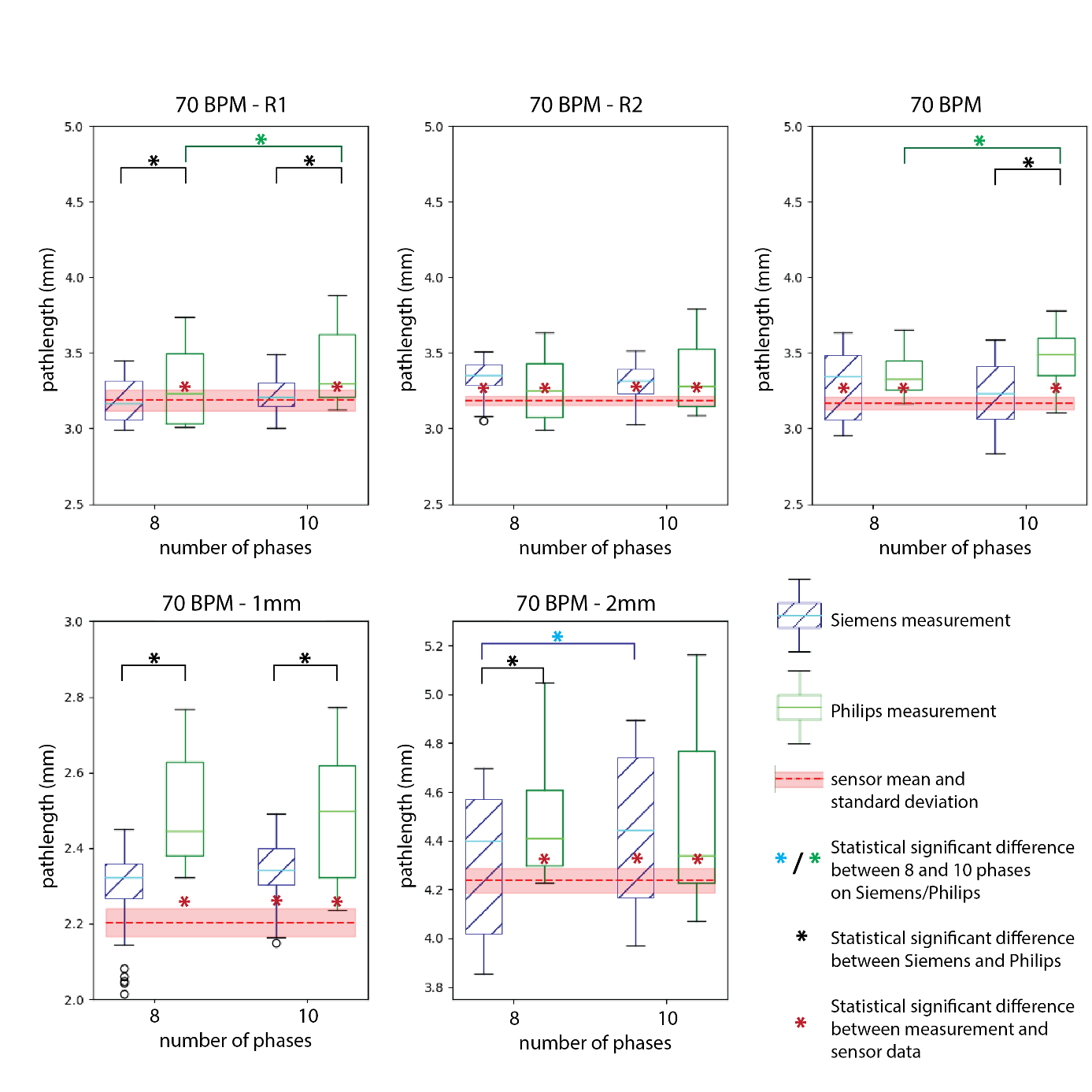
 ***Figure S.C2 –*** *Boxplots of the pathlength measurements for the Siemens (blue) and Philips (green) scans, reconstructed to either 8 or 10 cardiac phases, for variations to the baseline measurement at 70 beats per minute (BPM) with a motion amplitude of 1.5 mm: 2 repeated measurements (R1 and R2) and amplitude variation (1.0 mm and 2.0 mm). The mean (dashed red line) and standard deviation (light red band) are shown for each measurement as well.*

***Table S.C1 –*** *Overall comparison of the computed tomography (CT) data vs. the sensor and the baseline measurement vs. the repeated measurements. The comparisons are divided per scanner and per reconstruction and are shown for all measurements combined. The measurements include the amplitudes in z-direction (cranio-caudal direction, z-amplitude) and pathlengths. For the 3D measurements only the pathlengths are compared between the CT and sensor data.*

| **CT vs. sensor** | | | | | | |
| --- | --- | --- | --- | --- | --- | --- |
|  | | **Siemens^a^** | **Philips^a^** | **8 phases^b^** | **10 phases^b^** | **All measurements** |
| *z-amplitude (mm)* | | 0.01  (-0.02 to 0.05, p=1.000) | 0.07  (0.03 to 0.11, **p<0.001**) | 0.03  (0.00 to 0.06, **p=0.037)** | 0.05  (0.02 to 0.08, **p<0.001**) | 0.04  (0.03 to 0.04, **p=0.002**) |
| *pathlengths (mm)* | | 0.07  (-0.02 to 0.15, p=0.220) | 0.22  (0.13 to 0.30, **p<0.001**) | 0.10  (0.04 to 0.17, **p<0.001**) | 0.18  (0.12 to 0.24, **p<0.001**) | 0.14  (0.13 to 0.15, **p<0.001**) |
| 3D pathlengths (mm) | | 0.10  (0.01 to 0.19, **p=0.022**) | -0.03  (-0.12 to 0.07, p=1.000) | -0.08  (-0.14 to -0.01, **p=0.007**) | 0.15  (0.09 to 0.21, **p<0.001)** | 0.06  (0.03 to 0.08, **p<0.001**) |
| **Repeated measurements** | | | | | | |
| *z-amplitude (mm)* | *70 bpm vs. 70 bpm-R1* | -0.02  (-0.07 to 0.04, p=1.000) | -0.03  (-0.08 to 0.01, p=1.000) | -0.04  (-0.09 to 0.01, p=0.339) | -0.01  (-0.06 to 0.04, p=1.000) | -0.02  (-0.06 to 0.01, p=1.000) |
|  | *70 bpm vs. 70 bpm-R2* | 0.04  (-0.02 to 0.09, p=1.000) | -0.01  (-0.06 to 0.03, p=1.000) | 0.01  (-0.00 to 0.10, p=1.000) | 0.02  (-0.03 to 0.07, p=1.000) | 0.01  (-0.02 to 0.05, p=1.000) |
| *pathlengths (mm)* | *70 bpm vs. 70 bpm-R1* | -0.06  (-0.17 to 0.05, p=1.000) | -0.08  (-0.20 to 0.04, p=1.000) | -0.09  (-0.21 to 0.03, p=0.523) | -0.04  (-0.16 to 0.08, p=1.000) | -0.07  (-0.15 to 0.02, p=0.720) |
|  | *70 bpm vs. 70 bpm-R2* | 0.06  (-0.05 to 0.17, p=1.000) | -0.11  (-0.23 to 0.01, p=0.183) | -0.02  (-0.143 to 0.10, p=1.000) | -0.03  (-0.15 to 0.09, p=1.000) | -0.02  (-0.11 to 0.07, p=1.000 |

^a^ all measurements on the 8 and 10 phases reconstructions combined for the according scanner.

^b^ all measurements on the Siemens and Philips scanner combined for the according number of reconstructions.

**Table S.C2 –** Z-amplitude differences between the mean sensor data and the 8 and 10 phases reconstructions on the Siemens and Philips acquired ECG-gated CT scans.

| Measurement | phases | Siemens | | Philips | | z-amplitude Siemens vs. Philips ** – mm (95% CI, p) |
| --- | --- | --- | --- | --- | --- | --- |
|  |  | z-amplitude calculation vs. sensor – mm (95% CI, p) | z-amplitude  8 vs. 10 phases* – mm (95% CI, p) | z-amplitude calculation vs. sensor – mm (95% CI, p) | z-amplitude  8 vs. 10 phases* – mm (95% CI, p) |  |
| 50 bpm | 8 | -0.14 (-0.18 to -0.10, **p<0.001**) | 0.27 (0.23 to 0.31, **p<0.001**) | 0.03 (-0.01 to 0.07, p=0.195) | 0.02 (-0.06 to 0.01, p=1.000) | 0.17 (0.13 to 0.21, **p<0.001**) |
|  | 10 | 0.13 (0.10 to 0.17, **p<0.001**) |  | 0.01 (-0.03 to 0.05, p=1.000) |  | 0.15 (0.11 to 0.19, **p<0.001**) |
| 60 bpm | 8 | 0.02 (-0.02 to 0.06, p=1.000) | 0.03 (-0.06 to 0.01, p=0.353) | -0.02 (-0.05 to 0.02, p=1.000) | -0.01 (-0.05 to 0.03, p=1.000) | -0.04 (-0.08 to -0.00, **p=0.028**) |
|  | 10 | -0.01 (-0.04 to 0.03, p=1.000) |  | -0.03 (-0.06 to 0.01, p=0.526) |  | -0.02 (-0.06 to 0.02, p=1.000) |
| 70 bpm | 8 | 0.01 (-0.03 to 0.06, p=1.000) | -0.03 (-0.07 to 0.02, p=0.868) | 0.06 (0.02 to 0.10, **p=0.001**) | 0.02 (-0.03 to 0.06, p=1.000) | 0.04 (-0.01 to 0.08, p=0.180) |
|  | 10 | -0.02 (-0.06 to 0.03, p=1.000) |  | 0.07 (0.03 to 0.12, **p<0.001**) |  | 0.08 (0.04 to 0.12, **p<0.001**) |
| 70 bpm – R1 | 8 | -0.04 (-0.08 to 0.01, p=0.304) | 0.04 (-0.01 to 0.09, p=0.242) | 0.03 (-0.02 to 0.07. p=1.000) | 0.02 (-0.03 to 0.06, p=1.000 | 0.05 (0.01 to 0.10, **p=0.013**) |
|  | 10 | 0.00 (-0.05 to 0.05, p=1.000) |  | 0.04 (-0.01 to 0.09, p=0.132) |  | 0.03 (-0.02 to 0.08, p=0.826) |
| 70 bpm – R2 | 8 | 0.03 (-0.02 to 0.08, p=0.815) | -0.00 (-0.04 to 0.05, p=1.000) | 0.07 (0.01 to 0.11, **p<0.001**) | -0.00 (-0.05 to 0.05, p=1.000) | 0.01 (-0.03 to 0.06, p=1.000) |
|  | 10 | 0.04 (-0.01 to 0.09, p=0.407) |  | 0.06 (0.01 to 0.11, **p=0.002**) |  | 0.01 (-0.04 to 0.06, p=1.000) |
| 70 bpm – 1mm | 8 | 0.03 (0.00 to 0.07, **p=0.040**) | 0.01 (-0.02 to 0.04, p=1.000) | 0.10 (0.07 to 0.13, **p<0.001**) | -0.01 (-0.05 to 0.02, p=1.000) | 0.06 (0.02 to 0.09, **p<0.000**) |
|  | 10 | 0.04 (-0.01 to 0.08, **p=0.002**) |  | 0.09 (0.05 to 0.12, **p<0.001**) |  | 0.03 (-0.00 to 0.07, p=0.095) |
| 70 bpm – 2mm | 8 | 0.04 (-0.03 to 0.11, p=1.000) | 0.06 (-0.01 to 0.13, p=0.186) | 0.10 (0.03 to 0.17, **p<0.001**) | -0.00 (-0.07 to 0.07, p=1.000) | 0.07 (0.00 to 0.14, **p=0.026**) |
|  | 10 | 0.10 (0.03to 0.16, **p<0.001**) |  | 0.10 (0.03 to 0.17, **p<0.001**) |  | 0.01 (-0.06 to 0.08, p=1.000) |
| 80 bpm | 8 | 0.00 (-0.05 to 0.05, p=1.000) | -0.01 (-0.06 to 0.04, p=1.000) | 0.16 (0.11 to 0.21, **p<0.001**) | 0.02 (-0.03 to 0.07, p=1.000 | 0.14 (0.09 to 0.19, **p<0.001**) |
|  | 10 | -0.01 (-0.06 to 0.04, p=1.000) |  | 0.18 (0.13 to 0.23, **p<0.001**) |  | 0.17 (0.12 to 0.22, **p<0.001**) |
| 90 bpm | 8 | -0.01 (-0.06 to 0.05, p=1.000) | -0.00 (-0.06 to 0.05, p=1.000) | 0.03 (-0.03 to 0.09, p=1.000) | 0.04 (-0.02 to 0.10, p=0.504) | 0.02 (-0.03 to 0.08, p=1.000) |
|  | 10 | -0.01 (-0.07 to 0.04, p=1.000) |  | 0.05 (-0.00 to 0.11, p=0.106) |  | 0.04 (-0.01 to 0.10, p=0.387) |

* positive value indicates that 10 phases reconstructions measured z-amplitudes are larger than 8 phases reconstruction measured z-amplitudes

** positive value indicates that z-amplitudes calculated on Philips data are larger than z-amplitudes calculated on Siemens

**Table S.C3 –** Pathlengths differences between the mean sensor data and the 8 and 10 phases reconstructions on the Siemens and Philips acquired ECG-gated CT scans.

| Measurement | Phases | Siemens | | Philips | | z-amplitude Siemens vs. Philips ** – mm (95% CI, p) |
| --- | --- | --- | --- | --- | --- | --- |
|  |  | z-amplitude calculation vs. sensor – mm (95% CI, p) | z-amplitude  8 vs. 10 phases* – mm (95% CI, p) | z-amplitude calculation vs. sensor – mm (95% CI, p) | z-amplitude  8 vs. 10 phases* – mm (95% CI, p) |  |
| 50 bpm | 8 | -0.28 (-0.41 to -0.16, **p<0.001**) | 0.65 (0.52 to 0.78, **p<0.001**) | 0.09 (-0.03 to 0.22, p=0.481) | 0.09 (-0.04 to 0.21, p=0.689) | 0.41 (0.28 to 0.53, **p<0.001**) |
|  | 10 | 0.37 (0.24 to 0.50, **p<0.001**) |  | 0.18 (0.05 to 0.31, **p<0.001**) |  | -0.16 (-0.29 to -0.03, **p=0.005**) |
| 60 bpm | 8 | 0.15 (0.07 to 0.24, **p<0.001**) | -0.10 (-0.18 to -0.02, **p=0.006**) | -0.03 (-0.11 to 0.06, p=1.000) | 0.08 (-0.00 to 0.17, p=0.051) | -0.21 (-0.29 to -0.13, **p<0.001**) |
|  | 10 | 0.05 (-0.03 to 0.14, p=0.887) |  | 0.06 (-0.03 to 0.14, p=0.0.724) |  | -0.03 (-0.11 to 0.06, p=1.000) |
| 70 bpm | 8 | 0.11 (0.02 to 0.20**, p=0.003**) | -0.05 (-0.14 to 0.04, p=1.000) | 0.20 (0.11 to 0.29, **p<0.001**) | 0.12 (-0.03 to 0.20, **p=0.002**) | 0.08 (-0.01 to 0.16, p=0.177) |
|  | 10 | -0.06 (-0.02 to 0.15, p=0.569) |  | 0.31 (0.23 to 0.40, **p<0.001**) |  | 0.24 (0.15 to 0.33, **p<0.001**) |
| 70 bpm – R1 | 8 | -0.03 (-0.13 to 0.07, p=1.000) | 0.04 (-0.05 to 0.14, p=1.000) | 0.13 (0.03 to 0.23. **p=0.002**) | 0.12 (0.03 to 0.22, **p=0.003** | 0.10 (0.00 to 0.19, **p=0.046**) |
|  | 10 | 0.01 (-0.16 to 0.04, p=1.000) |  | 0.25 (0.16 to 0.35, **p<0.001**) |  | 0.18 (0.08 to 0.28**, p<0.001**) |
| 70 bpm – R2 | 8 | 0.16 (0.07 to 0.24, **p<0.001**) | -0.04 (-0.12 to 0.05, p=1.000) | 0.09 (0.01 to 0.18, **p=0.018**) | 0.01 (-0.08 to 0.09, p=1.000) | -0.06 (-0.15 to 0.02, p=0.490) |
|  | 10 | 0.12 (0.03 to 0.20, **p<0.001**) |  | 0.17 (0.08 to 0.25, **p<0.001**) |  | 0.06 (-0.04 to 0.13, p=1.000) |
| 70 bpm – 1mm | 8 | -0.05 (0.11 to 0.01, p=0.356) | 0.05 (-0.01 to 0.11, p=0.356) | 0.32 (0.26 to 0.38, **p<0.001**) | -0.01 (-0.08 to 0.05, p=1.000) | 0.29 (0.14 to 0.27, **p<0.001**) |
|  | 10 | 0.13 (0.07 to 0.20, **p<0.001**) |  | 0.30 (0.24 to 0.37, **p<0.001**) |  | 0.14 (0.08 to 0.20, **p<0.001**) |
| 70 bpm – 2mm | 8 | 0.05 (-0.09 to 0.19, p=1.000) | 0.14 (-0.00 to 0.29, **p=0.041**) | 0.24 (0.10 to 0.39, **p<0.001**) | -0.01 (-0.15 to 0.13, p=1.000) | 0.19 (0.05 to 0.33, **p=0.001**) |
|  | 10 | 0.20 (0.05 to 0.34, **p<0.001**) |  | 0.24 (0.09 to 0.38, **p<0.001**) |  | 0.04 (-0.10 to 0.18, p=1.000) |
| 80 bpm | 8 | 0.04 (-0.13 to 0.21, p=1.000) | -0.03 (-0.19 to 0.14, p=1.000) | 0.56 (0.39 to 0.73, **p<0.001**) | 0.09 (-0.07 to 0.26, p=1.000 | 0.47 (0.30 to 0.64, **p<0.001**) |
|  | 10 | -0.01 (-0.15 to 0.18, p=1.000) |  | 0.65 (0.49 to 0.72, **p<0.001**) |  | 0.59 (0.42 to 0.76, **p<0.001**) |
| 90 bpm | 8 | -0.05 (-0.17 to 0.09, p=1.000) | -0.03 (-0.08 to 0.14, p=1.000) | 0.04 (-0.0083 to 0.16, p=1.000) | 0.06 (-0.06 to 0.18, p=1.000) | 0.03 (-0.08 to 0.15, p=1.000) |
|  | 10 | -0.02 (-0.14 to 0.10, p=1.000) |  | 0.10 (-0.02 to 0.22, p=0.197) |  | 0.06 (-0.06 to 0.18, p=1.000) |
| 70 bpm – 3D | 8 | -0.16 (-0.23 to -0.07, **p<0.001**) | 0.51 (0.43 to 0.58, **p<0.001)** | -0.00 (-0.08 to 0.07, p=1.000) | -0.04 (-0.12 to 0.03, p=1.000) |  |
|  | 10 | 0.35 (0.28 to 0.43, **p<0.001**) |  | -0.05 (-0.13 to 0.03, p=0.978) |  |  |
| 70 bpm – 3D R1 | 8 | -0.13 (-0.22 to -0.05, **p<0.001)** | 0.48 (-0.40 to 0.56, **p<0.001)** | 0.14 (0.05 to 0.21, **p<0.001)** | -0.14 (-0.22, -0.05, **p<0.001)** |  |
|  | 10 | 0.35 (0.26 to 0.43, **p<0.001)** |  | 0.000 (-0.08 to 0.08, p=1.000) |  |  |

* positive value indicates that 10 phases reconstructions measured z-amplitudes are larger than 8 phases reconstruction measured z-amplitudes

** positive value indicates that z-amplitudes calculated on Philips data are larger than z-amplitudes calculated on Siemens
